# The satisfaction of elderly people with elderly caring social organizations and its relationship with social support and anxiety during the COVID-19 pandemic: a cross-sectional study

**DOI:** 10.1186/s12889-023-15951-x

**Published:** 2023-06-21

**Authors:** Shuo Ding, Guoqing Liu, Fuqin Xu, Kai Ji, Lanlan Zhao, Xin Zheng, Otsen Benjamin, Zhengsheng Wang, Shufan Yang, Ren Chen

**Affiliations:** 1 School of Health Services Management, Anhui Medical University, Hefei, 230032 China; 2 Registrars’ Department, University of Cape Coast, Cape Coast, Ghana; 3 School of Computing, Engineering and Built Environment, Edinburgh Napier University, Edinburgh, UK; 4 Research Department of Orthopaedics and Musculoskeletal Science, University College London, UCL, London, UK

**Keywords:** Social support, Satisfaction, Anxiety, Elderly caring, Social organizations

## Abstract

**Background:**

With the deepening of China’s aging population, higher demands have been placed on the supply of elderly care services. As one of the main sources of providing elderly care services, the quality of service provided by elderly caring social organizations (SOs) directly affects the quality of life of the elderly. In recent years, mental health issues among the elderly have become increasingly prominent, especially with the onset of the COVID-19 pandemic. Necessitating the need to pay much more attention to the social support and mental health of this population. This study, therefore, explores the mediating role of institutional satisfaction between the social support and anxiety levels of elderly people in Chongqing’s elderly caring SOs.

**Method:**

This study employed a multi-stage stratified random sampling method to survey 1004 service recipients in elderly caring social organizations from July to August 2022. The self-made sociodemographic questionnaire, institutional satisfaction questionnaire, MSPSS, and GAD-7 were used to collect data on sociodemographic characteristics, institutional satisfaction, social support, and anxiety levels of older adults. Exploratory Factor Analysis and Cronbach’s alpha were used to test construct validity and scale reliability, respectively. Data features were described with One-Way Analysis of Variance, while Multiple Linear Regression and Structural Equation Modeling were used to evaluate relationships between social support, institutional satisfaction, and anxiety levels.

**Results:**

The average institutional satisfaction score for elderly people in elderly caring SOs was 48.14 ± 6.75. Specifically, the satisfaction score for environmental quality and the satisfaction score for service quality were 16.63 ± 2.56 and 31.52 ± 4.76, respectively. In terms of socio-demographic variables, the presence of visits from relatives, personal annual average income, and self-rated health status all have significant effects on anxiety. Elders who receive visits from relatives have lower levels of anxiety compared to those who do not. Personal annual average income and self-rated health status are negatively correlated with anxiety levels. Social support had significant positive effect on institutional satisfaction, while institutional satisfaction had significant negative effect on anxiety. Institutional satisfaction partially mediated the relationship between social support and anxiety.

**Conclusions:**

Our research demonstrates that improving the quality of organizational services in elderly caring SOs and increasing institutional satisfaction among the elders has significant potential for reducing anxiety levels among the elderly. Additionally, the social support by visits from family members cannot be overlooked. We encourage increasing the frequency of family visits through various means to enhance the support provided to elderly individuals.

**Supplementary Information:**

The online version contains supplementary material available at 10.1186/s12889-023-15951-x.

## Background

As the world’s largest developing country, China has the world’s largest elderly population [1]. The seventh national census in 2020 revealed that 18.7% of the country’s total population, or 264 million people, are aged 60 and above, while those aged 65 and above account for 13.5%, or 190 million people [2]. Confronted with such a serious problem of aging and a series of social problems arising from it, the issue of elderly care and their physical and mental health has increasingly garnered high attention from governments and various sectors of society. In China, the elderly, as a vulnerable group, are not only in a vulnerable position in terms of physical function and economic status, but also in a more vulnerable mental health [3–5]. Elderly people are a high-risk group for anxiety(c). As they age, the physical functions of elderly people gradually decline, and the impact of social role changes, the loss of loved ones, and other adverse events can cause anxiety in the elderly. A survey indicates that mental and neurological disorders affect over 20% of the elderly population aged 60 or older globally, with the most common conditions being dementia, major depressive disorder (MDD), and anxiety [6]. In particular, in China ,anxiety as a common mental disorder among elderly people, up to 22.1% of the elderly have anxiety symptoms and 6.8% of them have disorders due to anxiety [7]. In addition, a study by Creighton and others has found that older adults living in nursing homes or retirement apartments are more likely to experience anxiety compared to those living in the community [8].

Anxiety in older adults is associated with greater risk of comorbidities, cognitive decline, reduced quality of life, prolonged recovery, severe disability, and mortality [9–11]. In addition, the anxiety of older adults may be affected by the COVID-19 pandemic and its associated social distancing measures. Since December 2019, the world has faced the challenge of COVID-19, a highly contagious disease caused by the SARS-CoV-2 virus [12]. The elderly and people with chronic illnesses are at the greatest risk of severe symptoms and death. However, a recent study from Brazil found that isolation measures taken to avoid viral infections may affect the mental health of older adults, exacerbating levels of depression and anxiety [13]. According to the researchers, given the lack of a vaccine or targeted treatment for COVID-19, isolation measures are widely used [14]. However, long-term isolation can result in loneliness, rage and skepticism about the future. Making them vulnerable to stress. Together, these factors ultimately lead to a worsening of mental conditions in those who have or have not received professional treatment for depression and anxiety [15, 16].

Consideration of perceived social support is crucial in anxiety prevention and intervention strategies, as it has been recognized as a highly effective factor in reducing anxiety levels [17–19]. Usually, social support is considered to be “social resources that people believe are available or provided to them” [20].And the term “perceived social support” refers to an individual’s subjective assessment of the support they receive and their level of satisfaction with that support [17]. Previous studies have suggested that anxiety is negatively associated with social support, implying that low perceived social support may heighten anxiety levels [21, 22]. The level of institutional satisfaction among elderly people is an important indicator of the quality of care provided in nursing homes. It quantitatively measures their satisfaction with the elderly care institutions and services [23].In China, with the change of demographic structure and people’s concept of old age, as well as children working and settling abroad, more and more families choose to send their elderly to nursing home or apartment. Elderly care facilities come in three forms: those managed by government organizations, social organizations (SOs), and private investors, all of which provide services for older adults [22, 24, 25]. Specifically, SOs are non-profit organizations that provide social services for the elderly. They participate in elderly care services by providing community services, organizing volunteers, and establishing care facilities. Their goal is to improve the quality of life and well-being of the elderly by providing socialized care services and support[26]. As one of the main institutions providing senior care services, the quality of services provided by social organizations will undoubtedly have an important impact on the elderly people receiving the services. There has been a lack of research examining the satisfaction levels of elderly individuals residing in elderly care facilities, particularly in those operated by social organizations.

Although a number of studies have examined social support, institutional satisfaction, and depression, the combined effects of these factors on anxiety and the underlying mechanisms of action between each are not yet clear [22, 27, 28]. What is the relationship between social support and anxiety? Does social support have a positive effect on institutional satisfaction? Is there a negative relationship between institutional satisfaction and anxiety? Does institutional satisfaction moderate the relationship between social support and anxiety? We aim to explore the association between social support, institutional satisfaction of elderly residents, and their anxiety levels by addressing the above questions.

## Methods

### Study design and data collection

We conducted this transect survey in Chongqing in July and August 2022. Chongqing is located in the southwest of inland China, with a permanent resident population of 32.1243 million, including 7.0104 million people aged 60 and above, accounting for 21.87% [29]. The city is representative of China’s aging population. In this study, multistage stratified random sampling was used. In the first stage, we used a random sampling method to sample 26 districts in Chongqing and four districts (D, J, T and R) were selected for investigation and research. In the second stage, we cooperated with the local civil affairs department, 80 organizations were selected from the list of elderly caring SOs provided by the Civil Affairs Bureau for in-depth investigation. In the third stage, we organized investigators to conduct a survey on elderly people of 80 elderly caring SOs. All investigators consisted of graduate students from Anhui Medical University and Chongqing Medical University, and they received professional training before the start of the survey to improve their questioning skills and reduce investigator bias. Our researchers conducted face-to-face interviews with participants in a structured manner, with the support of nursing home staff. And informed consent was obtained from all respondents. People with limited physical mobility and those who could not fully understand the verbal explanation were excluded, and a final valid sample of 1004 was included.

### Measurement

#### Sociodemographic characteristics

The Sociodemographic Characteristics comprised age, gender, education level, average annual personal income(yuan), self-perceived health level, marital status and whether there are relatives visiting. We categorized age into three groups: <70, 70–79, and ≥ 80 years. Educational level was divided into five categories: no formal education, primary school, middle school, high school, and bachelor’s degree or higher. Average annual personal income(yuan) include less than 6500,6500 to 15000,15000 to 24000,24000 to 75,000 and more than 75,000. Self-perceived health level was divided into poor, general, good, very good, and excellent. Marital status was dichotomized as either married with a spouse or not married, including those who were divorced, unmarried, or widowed. In particular, when we surveyed the number of visits to relatives of elderly people in social organizations for the elderly, we found that there was a significant proportion of elderly people (more than 30%) who had no relatives to visit them or whose relatives never visited them. Therefore, We adjusted our survey scale to change the frequency of relatives visits to a binary variable: Whether there are relatives visiting.

#### Social support

The Multidimensional Scale of Perceived Social Support (MSPSS) was utilized to evaluate the degree of social support received by the clients [30]. This scale measures the individual’s perceived social support from family, friends, and significant others. Participants’ total scores are derived from the sum of the scores of the family, friends and significant others dimensions. Participants were scored according to a seven-point Likert scale response format from 1 (very strongly disagree) to 7 (very strongly agree). Each person’s score ranged from 12 to 84. Higher scores indicate higher levels of social support [31]. In this study, the total social support Cronbach’s α was 0.936, Cronbach’s α was found to be 0.963 for the family support subfield, 0.962 for the friend support subfield, 0.917 for the special person support subfield.

#### Anxiety

Screening and measuring anxiety status of older adults in the past two weeks using the Generalized Anxiety Disorder 7-item scale [32]. The GAD-7 was initially validated in 2010 among general hospital outpatients in mainland China and has since been proven reliable for use in various sample groups [33]. Participants’ anxiety levels were measured using a four-point Likert scale, which assessed their anxiety over a two-week period. Scores ranged from 0 (not at all) to 3 (almost every day), with higher scores indicating greater levels of anxiety. The GAD-7 scale was used to calculate participants’ scores, with a range of 0 to 21. In addition, Cronbach’s α of this scale in the current study was 0.893.

#### Institutional satisfaction

A self-made institutional satisfaction scale was used, using a five-point Likert scale. The measurement of satisfaction with elderly care institutions contains two dimensions: elderly people’s satisfaction with the environment of elderly care institutions and the service quality of elderly care institutions. The total score of the two dimensions added together is the overall satisfaction of elderly care institutions. Scores range from 1 (very dissatisfied) to 5 (very satisfied). The possible scores for each participant ranged from 12 to 60, with higher scores indicating that the older person was more satisfied with the facility. The reliability of the overall satisfaction scale was high, with a Cronbach’s α value of 0.905, and the two sub-dimensions also showed good internal consistency with Cronbach’s α values of 0.824 and 0.878, respectively, surpassing the recommended level of 0.7. The structural validity was confirmed by the Kaiser-Meyer-Olkin (KMO) measure of sampling adequacy, which was greater than 0.7, indicating an appropriate factor analysis[34]. Respectively, the KMOs of the two dimensions were 0.781 and 0.889, the values of Bartlett’s sphericity test were all less than 0.001. To assess the factor loading values, previous studies [35, 36] have suggested that values at or above 0.3 can be deemed acceptable, while values exceeding 0.55 can be deemed favorable. The loading values of all items corresponding to the dimensions in Table 1 were found to be greater than 0.55. Additionally, the total variances explained by each of the three scales were 66.081% and 55.734%, respectively, indicating acceptable and good construct validity based on previous research criteria.

**Table 1:** Factor Loadings and Cronbach’s α: Assessing Dimension Reliability Using Exploratory Factor Analysis

Items	Environmental satisfaction	Service Quality Satisfaction
**Are you satisfied with the indoor temperature and humidity?**	0.835	
**Are you satisfied with the interior light?**	0.865	
**Are you satisfied with the indoor noise?**	0.788	
**Are you satisfied with the greening of the elderly institutions?**	0.759	
**Are you satisfied with the quality of incoming and outgoing hospital services?**		0.787
**Are you satisfied with the quality of life care services?**		0.812
**Are you satisfied with the quality of the catering service?**		0.726
**Are you satisfied with the quality of cleaning and sanitation services?**		0.759
**Are you satisfied with the quality of the laundry service?**		0.730
**Are you satisfied with the quality of cultural and entertainment services?**		0.726
**Are you satisfied with the quality of your spiritual support services?**		0.761
**Are you satisfied with the quality of rehabilitation services?**		0.598
**Total variance explained**	66.081%	55.734%
**Cronbach’s α**	0.781	0.889

### Statistical analysis

Descriptive statistics were used to characterize the sample using SPSS 26.0 and Mplus 8.3. Continuous variables were reported as mean ± standard deviation, while categorical variables were reported as percentages (%).Next, univariate analysis using t-tests and one-way analysis of variance was performed with SPSS 26.0 to investigate the effects of basic sociodemographic variables on social support, institutional satisfaction, and anxiety levels among the elderly. For the third step, we used Pearson correlations to examine the associations among the variables mentioned above. For the fourth step, we used Mplus 8.3 to test the fitness of our hypothesized model using SEM. To enhance the credibility of the SEM results, this study employed multiple linear regression as a robustness test. Using the subscale scores of social support, institutional satisfaction, and anxiety as observed variables, their total scores were treated as latent variables. This serves to support our hypothesis regarding the mediation model.

## Results

### Demographic data

As shown in Table 2, a whole of 1004 elderly people participated in this survey. Among them, 541 (53.9%) were male and 463 (46.1%) were female, The majority of participants were aged eighty years and older (50.6%), and the education level was mostly uneducated and elementary school, with 347 (34.6%) and 393 (39.1%) respectively. The most participants (82.3%) reported being unmarried, divorced, or widowed.315 (31.4%) of elders had no family visits(counted after admission to the nursing home),The majority of elderly people have an average annual personal income of less than RMB 15,000 (61.5%),and very few seniors earn more than RMB 75,000 per year(4.9%).In addition, most older adults’ self-rated health status was general and good, 39.8% and 29.1% of the total, respectively.

**Table 2:** Descriptive results of the sample

Variables	N (%), Mean ± SD
**Gender**	
Male	541(53.9)
Female	463(46.1)
**Age (years)**	
< 70	183(18.2)
70–79	313(31.2)
≥ 80	508(50.6)
**Education**	
No formal education	347(34.6)
Primary School	393(39.1)
Middle School	138(13.7)
High School	82(8.2)
Bachelor’s degree and above	44.(4.4)
**Marital status**	
Married have spouses	178(17.7)
Divorced, widowed, or unmarried	826(82.3)
**Whether there are relatives visiting**	
No	315(31.4)
Yes	689(68.6)
**Average annual personal income(yuan)**	
<6500	360(35.9)
6500–15,000	257(25.6)
15,000–24,000	153(15.2)
24,000–75,000	185(18.4)
>75,000	49(4.9)
**self-perceived health level**	
Poor	191(19.0)
General	400(39.8)
Good	292(29.1)
Very good	104(10.4)
excellent	17(1.7)
**Social support**	57.38 ± 15.95
From family	19.28 ± 7.54
From friends	18.53 ± 6.66
From significant others	19.57 ± 5.09
**Institutional satisfaction(total)**	48.14 ± 6.75
Environmental satisfaction	16.63 ± 2.56
Service Satisfaction	31.52 ± 4.76
**Anxiety**	2.50 ± 3.58

The study population had a mean social support score of 57.38 ± 15.95, a mean institutional satisfaction score of 48.14 ± 6.75, and a mean anxiety score of 2.50 ± 3.58. The participants’ anxiety scores ranged from 0 to 19, with a mean of 2.50 and a median of 1.

### Univariate analysis and correlations between variables

According to the univariate results of Table 3, both relative visits and self-rated health levels had statistically significant impact on social support, anxiety levels, institutional satisfaction, environmental quality satisfaction, and service quality satisfaction. Women’s satisfaction with nursing homes is higher than men’s, this is mainly reflected in the fact that women score higher than men in terms of satisfaction with service quality. Elderly individuals who are married and have a spouse tend to report higher levels of satisfaction with the quality of their environment and the services they receive, compared to those who are divorced, unmarried, or widowed. Therefore, their overall satisfaction with the institution is also higher than the latter. Elders with an average annual personal income of RMB 15,000 and above perceive higher social support than those with incomes below RMB 15,000. The perceived social support of women was significantly higher than that of men. Different levels of anxiety exist among elderly people with different levels of health, and the results show that those in poorer health have the highest anxiety scores so they are more likely to experience anxiety.

**Table 3:** Univariate analysis among variables

		Social Support		Anxiety		Institutional satisfaction(total)		Environmental satisfaction		Service Quality Satisfaction
**Gender**										
Male		52.61 ± 16.32		2.61 ± 3.74		47.39 ± 6.86		16.50 ± 2.60		30.89 ± 4.85
Female		62.96 ± 13.54		2.37 ± 3.39		49.02 ± 6.53		16.77 ± 2.51		32.25 ± 4.55
t/F		-10.986		1.081		-3.833		-1.656		-4.556
P		< 0.001		0.280		< 0.001		0.098		< 0.001
**Age (years)**										
< 70		52.84 ± 16.13		2.88 ± 4.20		47.27 ± 6.95		16.50 ± 2.75		30.77 ± 4.93
70–79		55.87 ± 16.18		2.58 ± 3.60		48.11 ± 6.34		16.72 ± 2.41		31.38 ± 4.57
≥ 80		59.95 ± 15.28		2.31 ± 3.31		48.48 ± 6.91		16.63 ± 2.56		31.87 ± 4.79
t/F		15.878		1.850		2.195		0.439		3.842
P		< 0.001		0.158		0.112		0.645		0.022
**Education**										
No formal education		54.05 ± 16.23		2.80 ± 3.81		47.94 ± 7.01		16.42 ± 2.60		31.52 ± 4.93
Primary School		56.56 ± 15.51		2.32 ± 3.41		47.68 ± 6.47		16.57 ± 2.52		31.11 ± 4.59
Middle School		60.59 ± 16.07		2.04 ± 3.25		49.03 ± 6.51		17.02 ± 2.37		32.00 ± 4.69
High School		65.31 ± 13.77		2.76 ± 3.82		49.11 ± 7.47		16.88 ± 2.94		32.23 ± 5.01
Bachelor’s degree and above		66.11 ± 10.76		2.61 ± 3.69		49.32 ± 6.26		17.02 ± 2.34		32.30 ± 4.43
t/F		14.556		1.559		1.887		1.939		1.829
P		< 0.001		0.183		0.111		0.102		0.121
**Marital status**										
Divorced, widowed, or unmarried		55.88,15.91		2.54,3.63		47.70,6.66		16.47,2.55		31.22,4.72
Married have spouses		64.35,14.24		2.28,3.34		50.22,6.82		17.34,2.51		32.89,4.74
t/F		-7.050		0.911		-4.506		-4.113		-4.269
P		< 0.001		0.337		< 0.001		< 0.001		< 0.001
**Whether there are relatives visiting**										
No		46.06 ± 15.33		3.44 ± 4.29		46.37 ± 6.39		16.19 ± 2.54		30.18 ± 4.52
Yes		62.56 ± 13.35		2.06 ± 3.11		48.96 ± 6.77		16.83 ± 2.55		32.13 ± 4.74
t/F		-16.456		5.126		-5.733		-3.702		-6.254
P		< 0.001		< 0.001		< 0.001		< 0.001		< 0.001
**Average annual personal income(yuan)**										
< 6500		54.05 ± 16.23		2.80 ± 3.81		47.64 ± 7.00		16.42 ± 2.60		31.52 ± 4.93
6500–15,000		56.56 ± 15.51		2.31 ± 3.41		47.68 ± 6.47		16.57 ± 2.52		31.11 ± 4.59
15,000–24,000		60.59 ± 16.07		2.04 ± 3.25		49.03 ± 6.51		17.03 ± 2.37		32.00 ± 4.69
24,000–75,000		65.31 ± 13.77		2.76 ± 3.82		49.11 ± 7.48		16.88 ± 2.94		32.23 ± 5.01
> 75,000		66.11 ± 10.76		2.61 ± 3.69		49.32 ± 6.26		17.02 ± 2.34		32.30 ± 4.43
t/F		14.556		1.559		1.887		1.939		1.829
P		< 0.001		0.183		0.111		0.102		0.121
**Self-perceived health level**										
Poor		55.05 ± 16.45		4.32 ± 4.48		47.14 ± 7.09		16.27 ± 2.53		30.86 ± 5.12
General		57.12 ± 15.73		2.65 ± 3.64		47.66 ± 6.76		16.47 ± 2.62		31.95 ± 4.73
Good		59.81 ± 15.26		1.50 ± 2.59		49.14 ± 6.57		16.96 ± 2.52		32.18 ± 4.52
Very good		56.81 ± 15.93		1.66 ± 2.67		48.81 ± 6.46		16.90 ± 2.51		31.90 ± 4.76
Excellent		51.59 ± 22.22		0.76 ± 1.03		49.71 ± 5.43		16.94 ± 2.01		32.76 ± 3.77
t/F		3.363		22.485		3.666		2.919		3.258
P		0.010		< 0.001		0.006		0.020		0.011

Table 4 displays the correlations among social support, institutional satisfaction, and anxiety. The study found that social support was linked to lower levels of anxiety and higher satisfaction with nursing institutions among the elderly. Besides, elderly people’s overall satisfaction with elderly care facilities is negatively correlated with anxiety.

**Table 4:** The correlation among social support, institutional satisfaction, and anxiety

Variables		Social support		Institutional satisfaction		Anxiety
**Social support**						
**Institutional satisfaction**		0.475**				
**Anxiety**		-0.264**		-0.353**		

**P < 0.001

### Test of study models

**Fig. 1:**
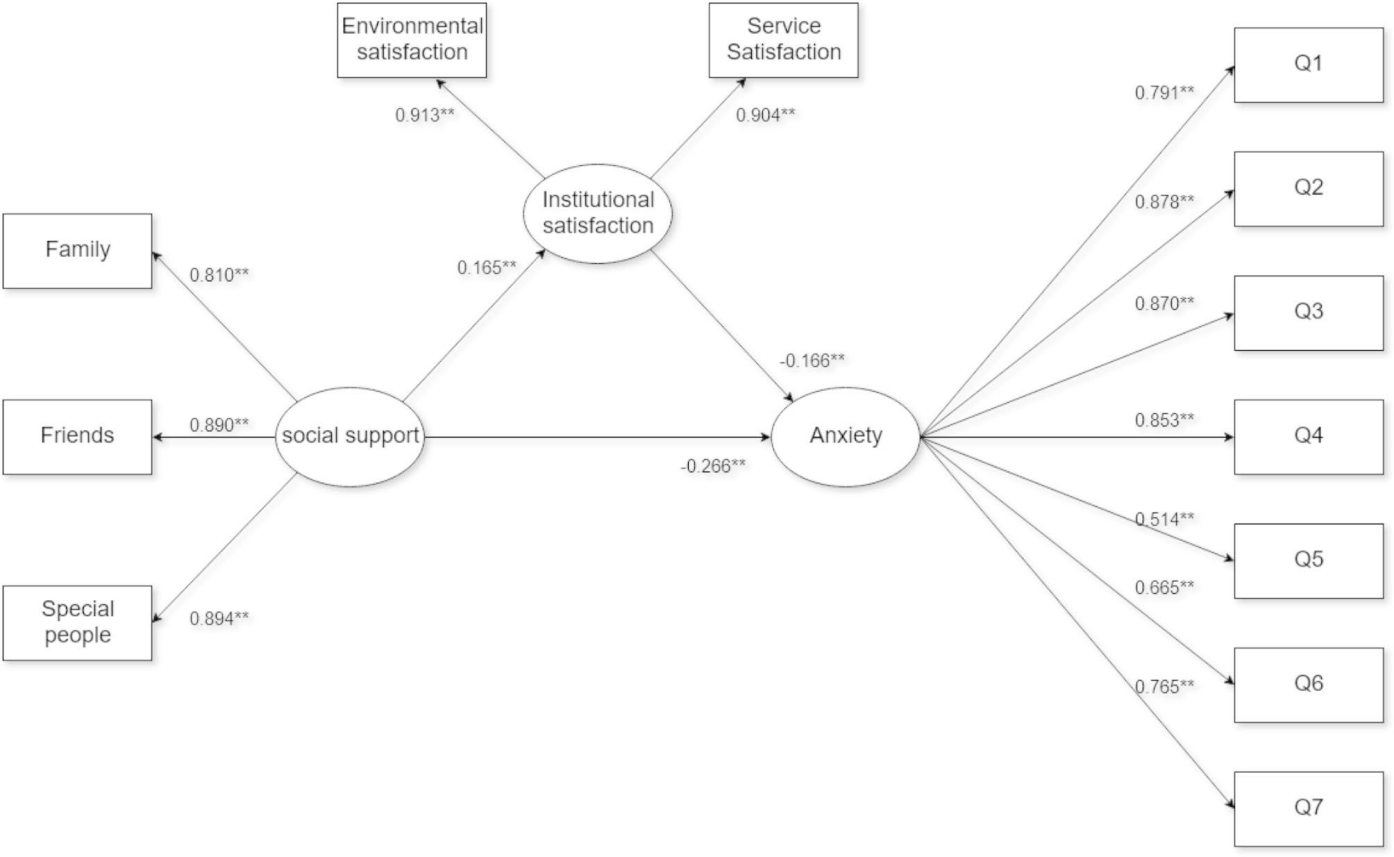
Structural equation model diagram.^**^P < 0.001

As shown in Fig. 1, the study employed SEM to quantify the associations among social support, institutional satisfaction, and anxiety. After incorporating socio-demographic characteristics into the model as covariates, the study determined that the coefficients and directions of each variable in the model remained constant, indicating that socio-demographic characteristics did not act as confounding factors, and were therefore excluded from the final model. The hypothetical model exhibited a good fit, as evidenced by the overall model fit indices: RMSEA = 0.063, SRMR = 0.035, CFI = 0.974, TLI = 0.966, SRMR = 0.035, and X^2^/df = 2.988. Figure 1 shows the results of the SEM analysis and the relationship between the variables. Social support was measured by three observed variables, support from special others contributed the most to perceived social support (coefficient = 0.894), and the other two observed variables, namely social support from family and social support from friends, were also highly correlated for the association of perceived social support. The correlation coefficients were 0.810 and 0.890, respectively. The anxiety status of older adults was measured by seven observed variables (Q1-Q7). The results from Fig. 1 address the four research questions regarding the relationship between social support, institutional satisfaction, and anxiety in the study. These findings were statistically significant, indicating strong evidence for these relationships. The study yielded noteworthy findings on the connections between social support, institutional satisfaction, and anxiety. Specifically, social support was found to have a beneficial negative impact on anxiety (coefficient = -0.266) and a beneficial positive impact on institutional satisfaction (coefficient = 0.165). Additionally, institutional satisfaction was found to have a beneficial negative impact on anxiety (coefficient = -0.166).Furthermore, The study also demonstrates the mediation hypothesis that we have proposed. The results revealed that institutional satisfaction played a significant mediating role in this relationship, with a 95% confidence interval ranging from − 0.138 to -0.018. All coefficients were statistically significant at P < 0.001. A summary of the direct, indirect, and total effects between social support, institutional satisfaction, and anxiety can be found in Table 5.

**Table 5:** The path coefficients in the hypothetical model

Model pathways		Standardized coefficient		95%C.I.
**Direct effects**				
Social support → Anxiety		-0.266		-0.374 to -0.152
Social support → Institutional satisfaction		0.165		0.080 to 0.0.229
Institutional satisfaction → Anxiety		-0.166		-0.289 to -0.037
**Indirect effects**				
Social support → Institutional satisfaction → Anxiety		-0.079		-0.138 to -0.018
**Total effects**				
Social support → anxiety		-0.346		-0.430 to -0.247

### Robustness check

Table 6 summarizes the results of our multiple linear regression model. The model employed anxiety as the dependent variable, and social support and institutional satisfaction as independent variables, while the sociodemographic variables were included in the model as controlling factors. Among the socio-demographic variables, the presence of visits from relatives, average annual personal income, and self-rated health level all had significant effects on anxiety. Elderly people who had visits from relatives showed lower levels of anxiety compared to those who did not have visits from relatives. Average annual personal income and self-rated health level were negatively associated with anxiety levels. In addition, Linear models showed that both social support and institutional satisfaction and anxiety had significant effects on anxiety (p < 0.001). After controlling for sociodemographic variables, the coefficients revealed that there was a negative association between anxiety and both social support (β = -0.028) and institutional satisfaction (β = -0.156). This implies that as social support and institutional satisfaction increase, anxiety levels decrease. The results are consistent with the SEM results and provide positive support for our SEM results.

**Table 6:** Multiple linear regression analysis of factors associated with anxiety

		Unstandardized				Standardized						95% CI for B		
Variables		B		SE		BETA		t		p		Lower limit		Upper limit
Age (years)														
< 70(ref)														
70–79		-0.276		0.318		-0.036		-0.866		0.387		-0.900		0.349
≥ 80		-0.573		0.308		-0.080		-1.857		0.064		-1.178		0.033
Gender														
Male(ref)														
Female		0.220		0.238		0.031		0.927		0.149		-0.246		0.686
Marital status														
Divorced, widowed, or unmarried(ref)														
Married have spouses		-0.060		0.294		-0.06		-0.206		0.837		-0.637		0.516
Whether there are relatives visiting														
No(ref)														
Yes		-1.455		0.257		-0.189		-5.664		< 0.001		-1.959		-0.951
Education														
No formal education (ref)														
Primary School		-0.332		0.253		-0.045		-1.313		0.190		-0.828		0.164
Middle School		-0.413		0.352		-0.040		-1.173		0.241		-1.104		0.278
High School		0.480		0.441		0.037		1.090		0.276		-0.385		1.346
Bachelor’s degree and above		0.677		0.579		0.039		1.169		0.243		-0.460		1.813
Average annual personal income(yuan)														
<6500(ref)														
6500–15,000		-0.821		0.282		-0.100		-2.916		0.004		-1.347		-0.268
15,000–24,000		-1.079		0.337		-0.108		-3.202		0.001		-1.741		-0.418
24,000–75,000		-1.021		0.333		-0.111		-3.063		0.002		-1.675		-0.367
>75,000		-1.710		0.561		-0.103		-3.048		0.002		-2.811		0.609
Health Status														
Poor(ref)														
General		-1.542		0.302		-0.211		-5.099		< 0.001		-2.135		-0.949
Good		-2.691		0.320		-0.341		-8.413		< 0.001		-3.318		-2.063
Very good		-2.584		0.417		-0.220		-6.197		< 0.001		-3.402		-1.766
excellent		-3.729		0.865		-0.134		-4.311		< 0.001		-5.426		-2.031
Social support		-0.028		0.007		-0.124		-3.722		< 0.001		-0.043		-0.013
Institutional satisfaction		-0.156		0.018		-0.294		-8.803		< 0.001		-0.181		-0.121

## Discussion

Previous studies have shown that anxiety has seriously affected the healthy life expectancy of the elderly, not only harming their physiology, but also impairing their mental health [37, 38]. Gay et al. demonstrated that anxiety can independently predict the occurrence of depressive symptoms [39]. The mental health of older adults requires special attention, especially with the presence of the COVID-19. In addition, with improved living conditions and advances in the healthcare system, the average life expectancy of Chinese people is increasing. In 1990, the mean life expectancy was 69.03 years, which increased to 75.01 years in 2010. It is anticipated that the average life expectancy will rise to nearly 80 years by 2050 [40]. The increase in average life expectancy has also placed greater demands on elderly care services. As part of the 14th Five-Year Plan, the Chinese government has emphasized the need to expand the supply of elderly care services and increase the allocation of elderly care facilities. This is in response to the growing demand for elderly care services in China due to the aging population [41]. In this context, elderly caring SOs are playing an increasingly important role [22, 42]. Hence, this study investigated how social support and institutional satisfaction are related to anxiety levels among elderly individuals residing in elderly caring SOs located in Chongqing during the COVID-19 pandemic.

### Current status and influential factors of anxiety among recipients of institutional services

The one-child policy and migration to urban areas have led to a situation where many elderly individuals lack care and support, and as a result, resorting to nursing homes. Some elderly people enter nursing homes voluntarily, while others are forced to enter. The traditional elderly care model in China is home-based care. A survey conducted in China found that out of 6,997 elderly people (89.1%) preferred to receive home-based care, while only 8.2% preferred institutional care [43]. One interesting finding was that one reason for their refusal to reside in elderly care institutions was concern for their family reputation. This is because the traditional Chinese concept of elderly care places the responsibility on adult children to care for their parents. Therefore, living in an elderly care institution may be perceived as a reflection of the children’s lack of filial piety. This cultural milieu exposes many elders to suffer from anxiety after moving into a nursing home. Previous studies have demonstrated the effectiveness of social support, as a useful resource for reducing psychological stress and alleviating symptoms such as depression and anxiety [44, 45]. In our SEM analysis, both social support and institutional satisfaction were negatively correlated with anxiety, consistent with prior research [22]. More meaningfully, we found that social support directly and indirectly negatively affected anxiety by positively affecting institutional satisfaction. Our investigations found that the availability of visits from relatives, average annual personal income and self-rated health status were all influential factors for anxiety.

### The mediating role of institutional satisfaction

Satisfaction with the institution was found to be significantly associated with lower levels of anxiety, which is similar to previous studies [22, 24]. On one hand, when elderly people are satisfied with elderly caring SOs, they may feel more supported and cared for, which can enhance their self-confidence, alleviate feeling of loneliness, and reduce anxiety. Specifically, their satisfaction with the institution can impact their overall satisfaction with life [46]. The quality of life of elders in elderly caring SOs is an important component of their satisfaction. Comfortable accommodations, healthy meals and life security can improve the quality of life for elders. If elderly people’s quality of life is assured, they may feel more reassured and content, which may reduce anxiety. Meanwhile, institutional satisfaction is also related to the self-esteem and self-confidence of older people. Elderly people may encounter various difficulties and challenges in elderly caring SOs, such as health problems and interpersonal issues. However, sufficient support and attention, may enhance their self-esteem and self-confidence, thus reducing anxiety.

On the other hand, institutional satisfaction is moderated by social support. Our research indicated that among the social support received by elderly people, support from significant others was the highest. This was expected as the baseline survey of this study showed that a vast majority of elderly people are divorced or widowed, so they could not receive support and comfort from their spouses. During the COVID-19, the implementation of isolation policies made it more difficult for the elderly to meet with their families and friends, so they felt less support from their loved ones. Nevertheless, the closed environment of the care institutions made the contact between caregivers and the elderly more frequent, thereby influencing caregivers to naturally assume the roles of family and friends in the elderly care delivery process. The quality of service and attitude of caregivers directly affect the satisfaction of the elderly with the institution.

### The relationship between anxiety and family visitation, income, and self-reported health

Elderly people in care institutions who received visits from family members showed lower levels of anxiety compared to those who had never been visited by relatives. These results align with those of a prior study [47]. Visits by from family members can be seen as a demonstration of care, and to an extent, a form of social support. As a result, elderly individuals who are frequently visited by their family members receive higher levels of social support to fight anxiety. The frequency of visits from family members is indicative of the level of social support received by the elderly. As direct descendants, and important sources of financial and emotional support for the elderly, children, play a significant role as caregivers.

Similar to previous studies [48, 49], our findings indicate that there is an inverse relationship between average annual income and anxiety scores among older adults. Specifically, those with higher income tend to exhibit lower levels of anxiety. This may be because elders with higher disposable income are likely to have better material conditions and higher social status, factors that can lead to a higher sense of security and self-esteem. This sense of stability and satisfaction can reduce anxiety. In addition, elders with higher incomes are also likely to have more social opportunities and social support, which leads to more emotional support and practical help, making the elderly feel cared for and valued.

Elders who self-reported good health had significantly lower levels of anxiety than those with poor health. This may be related to their chronic disease status, as most older adults in China have one or more chronic diseases. According to a survey, the prevalence of chronic diseases among urban and rural elderly people in China was nearly 80% in 2015, of which the prevalence of coexisting chronic diseases was 48.8% [40]. Previous studies have revealed that chronic disease and comorbid chronic diseases significantly increase anxiety levels [50–52]. Elderly people may face more physical and psychological burdens that affect their quality of life [53].

### Countermeasures and suggestions

The findings of our study suggest that social support may impact the anxiety levels of elderly individuals indirectly, by affecting their satisfaction with elderly care service providers. Elderly individuals who feel supported by their families and nursing staff are more likely to be satisfied and enjoy living in nursing homes. These positive experiences and feelings may alleviate the anxiety of the elderly. Therefore, regarding the research model, we propose the following : firstly, we should focus on improving the service quality of elderly caring SOs, provide training for caregivers, fully consider the personalized needs and differentiated characteristics of the elderly, and pay attention to maintaining the privacy and dignity of the elderly in daily care processes. Implement a people-oriented nursing concept. At the same time, strengthen the management and supervision of elderly caring SOs to ensure the quality and effectiveness of services. Secondly, integrate service measures, seek cooperation from the government, medical institutions, and other social organizations, and gradually establish a medical-nursing integrated care model that combines medical services, daily care, social activities, and security. Provide comprehensive medical and life care services for the elderly, meet their various healthcare needs, socializing, culture, and entertainment, and improve their quality of life and health level. Finally, it is important to value the role of family involvement in the care of elderly people. Visits from family members can increase the sense of security and quality of life of elderly people, and reduce their levels of anxiety. Typically, elderly caring SOs should provide convenient visiting conditions for family members, and although the COVID-19 still exists, this should not be an obstacle to family visits. When family members have health certificates (such as nucleic acid test reports), every effort should be made to reduce obstacles to their visiting process. Special attention should be given to elderly people who do not receive visits from relatives to improve the elderly’s well-being. Caregivers should communicate and share information with family members who are unable to visit regularly. This helps keep family members informed of the elderly person’s condition and promotes better communication between relatives and the elderly. Elderly caring SOs should make full use of Internet technology to build online communication platforms, allowing the elderly and their families to contact and communicate at any time through methods such as video calls and online chats, reducing the impact of epidemic prevention policies, geographical location, and time on family visits.

This study has several limitations. Being a cross-sectional study, it cannot establish causal relationships between different factors. Secondly, the satisfaction of elderly individuals with elderly care service providers was measured using a self-designed survey questionnaire, which although demonstrated acceptable reliability and validity, may still have some limitations. Lastly, our study sample was limited to Chongqing, and thus, caution should be exercised when generalizing our findings to other regions of the country.

## Conclusions

Our study shows that social support and anxiety are indirectly related, with institutional satisfaction playing a mediating role. The higher the level of social support received, the higher the institutional satisfaction of elders. Higher institutional satisfaction can significantly reduce anxiety levels. Therefore, improving elders’ satisfaction with elderly caring SOs is an effective measure to reduce their anxiety levels. In addition, factors such as visitation by relatives, personal annual income, and self-rated health status are associated with levels of anxiety among the elderly in care institutions.

## Electronic supplementary material

Below is the link to the electronic supplementary material.

Supplementary Material 1

## Data Availability

The datasets used during the current study are available from the corresponding author on reasonable request.

## Abbreviations

SOs: Social Organizations
