# Supplementary material for: The satisfaction of elderly people with elderly caring social organizations and its relationship with social support and anxiety during the COVID-19 pandemic: a cross-sectional study
Source: BMC Public Health. 2023 Jun 21;23:1206. doi: 10.1186/s12889-023-15951-x (PMC10286353; doi:10.1186/s12889-023-15951-x)
Supplement: Supplementary file 1 — Supplementary Material 1 [file 12889_2023_15951_MOESM1_ESM.docx]

| **Satisfaction of the elderly with the environment of elderly care institutions.** | | | | | |
| --- | --- | --- | --- | --- | --- |
| **Item** | ① | ② | ③ | ④ | ⑤ |
| **Are you satisfied with the indoor temperature and humidity?** |  |  |  |  |  |
| **Are you satisfied with the interior light?** |  |  |  |  |  |
| **Are you satisfied with the indoor noise?** |  |  |  |  |  |
| **Are you satisfied with the greening of the elderly institutions?** |  |  |  |  |  |

| **Satisfaction of the elderly with the service quality of elderly care institutions.** | | | | | |
| --- | --- | --- | --- | --- | --- |
| **Item** | ① | ② | ③ | ④ | ⑤ |
| **Are you satisfied with the quality of incoming and outgoing hospital services?** |  |  |  |  |  |
| **Are you satisfied with the quality of life care services?** |  |  |  |  |  |
| **Are you satisfied with the quality of the catering service?** |  |  |  |  |  |
| **Are you satisfied with the quality of cleaning and sanitation services?** |  |  |  |  |  |
| **Are you satisfied with the quality of the laundry service?** |  |  |  |  |  |
| **Are you satisfied with the quality of cultural and entertainment services?** |  |  |  |  |  |
| **Are you satisfied with the quality of your spiritual support services?** |  |  |  |  |  |
| **Are you satisfied with the quality of rehabilitation services?** |  |  |  |  |  |

note：①Very Dissatisfied；②Dissatisfied；③Neutral；④Satisfied；⑤Very Satisfied
